# Six-Minute Walk Distance and Long-Term Outcomes in Contemporary Left Ventricular Assist Device Recipients: The ELEVATE Registry

**DOI:** 10.1093/icvts/ivag241

**Published:** 2026-09-11

**Authors:** William Herrik Nielsen, Finn Gustafsson, Steven Shaw, Jens Garbade, Michiel Morshuis, Daniel Zimpfer, Jacob Lavee, Laura Walker, Carlye Kraemer, Carlo Gazzola, Diyar Saeed, ELEVATE Registry Investigators

**Affiliations:** Department of Cardiology, Copenhagen University Hospital, Rigshospitalet, Copenhagen 2100, Denmark; Department of Clinical Medicine, University of Copenhagen, Copenhagen 2200, Denmark; Heart Failure Division, Abbott Cardiovascular, Abbott Park, IL 60064, United States; The Transplant Centre, Manchester University NHS Foundation Trust, Wythenshawe Hospital, Manchester M23 9LT, United Kingdom; Department of Cardiothoracic Surgery, Klinikum Links der Weser, Bremen 28277, Germany; Department of Cardiothoracic Surgery, Herz- und Diabeteszentrum NRW, Bad Oeynhausen 32545, Germany; Department of Surgery, Division of Cardiac Surgery, Medical University of Vienna, Vienna 1090, Austria; Department of Cardiac Surgery, Sheba Medical Center, Tel Aviv 5265601, Israel; Faculty of Medical and Health Sciences, Tel Aviv University, Tel Aviv 6997801, Israel; Heart Failure Division, Abbott Cardiovascular, Abbott Park, IL 60064, United States; Heart Failure Division, Abbott Cardiovascular, Abbott Park, IL 60064, United States; Heart Failure Division, Abbott Cardiovascular, Abbott Park, IL 60064, United States; Heart Center Niederrhein, Health and Medical University Krefeld, Krefeld 47805, Germany

**Keywords:** adverse events, LVAD, prognosis, six-minute walk distance, 6MWD

## Abstract

**Objectives:**

This study aimed to assess the prognostic value of six-minute walk distance (6MWD) in stable HeartMate 3 (HM3) recipients.

**Methods:**

Data from patients with available 6MWD at 6 months post-implant were obtained from the prospective, multicentre ELEVATE registry. Overall survival and adverse event rates were analysed using Cox regression models adjusted for age and sex, with 6MWD as a continuous predictor, and stratified by median distance (350 m).

**Results:**

Six-minute walk distance at 6 months was available in 194 HM3 recipients. Patients walking < median were older (57 vs 52 years, *P* = .002), had more comorbidities, and reported lower quality of life. Six-minute walk distance correlated positively with haemoglobin (*r* = 0.20, *P* = .006) and EuroQoL-5 Dimensions visual analogue scale (EQ-5D VAS) (*r* = 0.23, *P* = .004), and inversely with New York Heart Association (NYHA) class (*r* = –0.47, *P* < .0001). Kaplan-Meier analyses of patients walking < or ≥ 350 m showed no significant differences in survival (log-rank *P* = .379), right heart failure (HF) (*P* = .334), or rehospitalization (*P* = .243), while survival free from haemocompatibility-related adverse events differed significantly, although marginally (*P* = .049). Cox models confirmed that 6MWD had no independent prognostic value for mortality (HR 0.98, 95% CI, 0.96-1.01, *P* = .26) or secondary outcomes. However, patients with < median 6MWD experienced higher rates of major bleeding (Incidence rate ratio (IRR) 2.88, *P* < .001), major infection (IRR 2.14, *P* < .0001), and renal dysfunction (IRR 4.78, *P* = .033).

**Conclusions:**

Shorter 6MWD 6 months after HM3 implantation was associated with a higher risk of some adverse events (AEs) but was not prognostic for mortality, HF recurrence, or rehospitalization risk. The prognostic value of 6MWD in HF patients without circulatory support cannot be extrapolated to patients treated with current left ventricular assist devices (LVADs).

**Clinical trial registration number:**

ClinicalTrials.gov Identifier: NCT02497950.

## INTRODUCTION

Outcomes following left ventricular assist device (LVAD) implantation have been improving in recent years, with median survival exceeding 5 years and decreasing adverse event rates.^1^^,^^2^ Furthermore, patients stay on support for longer than in previous eras.^3^ With a growing population of long-term LVAD patients comes a need for prognostic markers for individualized management of patients in a manner similar to that of heart failure (HF) patients not receiving mechanical circulatory support. Six-minute walk distance (6MWD) is a well-established and robust predictor of clinical outcomes in patients with HF.^4–7^ Six-minute walk distance provides a practical, submaximal measure of functional capacity and is associated with patient-centred outcomes such as quality of life, exercise tolerance,^8^^,^^9^ and predicting hospitalization and mortality risk.

In advanced HF patients supported with an LVAD, functional assessment by cardiopulmonary exercise testing and/or 6MWD as a routine procedure during routine follow-up is recommended by the International Society for Heart & Lung Transplantation (ISHLT) (class IIa); however, evidence supporting its utility remains limited (level C).^10^ While peak oxygen uptake (pVO_2_) is a strong prognostic marker of mortality in LVAD patients,^11^^,^^12^ the predictive value of 6MWD has not been investigated in contemporary, centrifugal-flow devices. With very limited information on the prognostic value of 6MWD in LVAD recipients^13^ and given that the relationship between submaximal (6MWD) and maximal (pVO_2_) exercise capacity appears less tightly connected in LVAD patients, compared with non-LVAD HF patients,^14–16^ the aim of this study was to assess the prognostic significance of 6MWD on long-term outcomes in chronic, ambulatory HeartMate 3 (HM3) recipients. Specifically, we tested the hypothesis that longer 6MWD 6 months after HM3 implantation is associated with better survival and fewer adverse events during long-term follow-up.

## METHODS

This analysis used data from the ELEVATE registry (Evaluating the HeartMate 3 with Fully Magnetically Levitated Technology in a Post-Market Approval Setting; ClinicalTrials.gov Identifier: NCT02497950), a prospective international post-market registry of patients receiving the HM3 LVAD after regional commercial approval in Europe, Singapore, and Israel (following CE Mark approval in October 2015) and Kazakhstan (January 2015). The registry design, inclusion criteria, follow-up procedures, and primary results have been published previously.^2^^,^^17–20^ The registry was conducted in accordance with the Declaration of Helsinki and approved by the ethics committee at each participating institution. Patients who provided written informed consent were consecutively enrolled from March 2015 to February 2017; only those who declined participation were excluded from the registry. While patients with a primary LVAD implantation and those undergoing a pump replacement from another durable LVAD were enrolled, the present analysis included only de novo implants, as clinical profiles differ considerably between these 2 groups. Patients from the anonymized ELEVATE cohort^2^ were excluded from the analysis because functional capacity data were not available.

Patients were initially followed for 24 months post-implant or until an outcome (heart transplantation, explantation, death, or withdrawal, whichever occurred first). After the initial follow-up period ended in February 2019, the ELEVATE registry was extended to 5 years, and 247 subjects provided written consent to participate in the long-term follow-up. Postoperative care followed local *standard of care* practices at each participating centre rather than a prespecified registry protocol. Baseline variables comprised patient demographics, comorbidities (including prior cardiovascular disease), echocardiographic and haemodynamic measurements, New York Heart Association (NYHA) functional class, and INTERMACS profile. Functional capacity was assessed using the 6MWD, and health-related quality of life was assessed using the EuroQoL-5 Dimensions (EQ-5D) questionnaire. Clinical and functional follow-up was performed at 6 months and annually from 1 to 5 years after implantation. Outcomes and adverse events were classified according to INTERMACS definitions, consistent with prior HM3 studies.^1^^,^^2^ Haemocompatibility-related adverse events (HRAEs) were defined as a composite of non-surgical bleeding, stroke, or pump thrombosis. Although data were not monitored by on-site visits, entries were reviewed remotely and queries were issued for missing or inconsistent data.

For this analysis, patients with an available 6MWD at the 6-month visit were stratified into 2 groups, based on their ability to walk more or less than the collective median distance at 6 months. Baseline data and adverse events at any time after 6 months up to 5 years are reported, as well as overall survival, survival free from HRAE, any-cause rehospitalizations after 2 years of follow-up, and survival free from right HF (RHF). A regression analysis was performed to determine whether the 6MWD at 6 months was prognostic of survival outcomes, after adjusting for age and sex. Also, linear correlations between 6MWD and NYHA class, haemoglobin, and EQ-5D visual analogue scale (VAS) score were investigated. The primary outcome measure was overall 5-year survival based on 6MWD 6 months post-implant. Secondary outcomes included survival free from adverse events, differences in adverse event rates between groups, and correlates of 6MWD at 6 months.

### Statistical analysis

Continuous variables are summarized as mean ± SD unless stated otherwise, whereas categorical variables are summarized as counts and percentages. Time-to-event outcomes were analysed using Kaplan-Meier estimates and Cox proportional hazards models. Patients were censored at heart transplantation, explantation, withdrawal, or at the last available follow-up. Adverse events are reported both as the proportion of affected patients and event rates per patient year (EPPY). Missing data were not imputed, except for 6MWD: patients who were unable to perform the test due to HF symptoms were assigned a distance of 0 m; otherwise, missing 6MWD data were not imputed. Regression analyses were performed using a multivariable Cox regression, and Pearson’s correlation was used to assess the linear relationship between distance walked and other variables. Statistical analysis was performed using SAS version 9.4.

## RESULTS

Of the 418 patients who were followed for at least 6 months post-implant, 6MWD was available in 194 (46%) patients. Forty-four patients (23%) walked less than 300 m, of whom 8 patients (4%) were unable to perform the 6MWD due to HF symptoms, resulting in an imputed 6MWD of 0 m. The distribution of 6MWD at 6 months is shown in **Figure S1**. Overall, patient characteristics at the 6-month follow-up included an average age of 54 years, a mean body mass index of 27.5 kg/m^2^, 86% males, and 27% implanted with a destination intent. Atrial fibrillation was the most common comorbidity (43%), followed by diabetes (28%) and chronic obstructive pulmonary disease (14%). The included cohort was generally comparable to the ELEVATE registry patients not included in this analysis because of missing 6MWD at 6 months, but some differences were found (**Table S1**). Compared with patients without, patients with an available 6MWD at 6 months were younger, had better renal function, slightly higher blood pressure, better NYHA class, lower filling pressures (at pre-implant right heart catheterization), and walked further at the 6MWD at the pre-implant evaluation. Still, long-term survival did not differ between patients with or without a 6MWD at 6 months (**Figure S2**).

Patient characteristics, stratified by 6MWD at 6 months, can be found in **Table 1**. Patients who walked less than the median distance (350 m) at 6 months had more comorbidities, were older (57 vs 52 years, *P* = .002), reported worse quality of life (EQ-5D VAS score 65 ± 16 vs 71 ± 17, *P* = .03), and were less likely to be in NYHA class I or II (70% vs 90%, *P* = .002). There was no difference in left ventricle ejection fraction (*P* = .625), HF aetiology (*P* = .709), or body mass index (*P* = .937). At 6 months, 6MWD demonstrated a weak but statistically significant positive correlation with both quality of life and haemoglobin levels. Specifically, higher 6MWD was associated with higher EQ-5D VAS scores (*r* = 0.23, *P* = .004, **Figure S3**) and higher haemoglobin concentration (*r* = 0.20, *P* = .006, **Figure S4**). Furthermore, 6MWD showed a moderate negative correlation with NYHA class (*r* = –0.47, *P* < .0001, **Figure S5**).

**Table 1.** Patient Characteristics, Stratified by Distance Walked </≥ Median (350 m) at 6 Months Post-Implant

	Distance < median (*n* = 89)	Distance ≥ median (*n* = 105)	*P*
Age (years)	56.9 ± 11.0	51.5 ± 12.8	.002
Female sex	17 (19.1%)	10 (9.5%)	.055
Caucasian	68 (77.3%)	77 (73.3%)	.528
Body mass index (kg/m^2^)	27.9 ± 5.2	27.8 ± 4.7	.938
Ischaemic heart failure aetiology^a^	40 (44.9%)	50 (47.6%)	.710
Destination intent	29 (32.6%)	24 (22.9%)	.147
INTERMACS profile 1 or 2	31 (35.2%)	19 (18.4%)	.013
NYHA class I or II	54 (70.1%) [*n *= 77]	78 (89.7%) [*n* = 87]	.001
6MWD pre-implant	105.7 ± 129.0 [*n* = 72]	166.1 ± 155.1 [*n* = 98]	.008
6MWD at 6 months	243 ± 106	445 ± 113	–
EQ-5D VAS score	65 ± 16	71 ± 17	.034
LVEF (%)	22.5 ± 8.3	21.9 ± 8.4	.625
Comorbidities			
History of atrial fibrillation^b^	47 (52.8%)	37 (35.2%)	.014
History of stroke^b^	8 (9%)	12 (11.4%)	.578
History of right heart failure^c^	13 (14.6%)	5 (4.8%)	.019
History of gastrointestinal bleeding^c^	2 (2.2%)	8 (7.6%)	.112
History of driveline infection^c^	11 (12.4%)	7 (6.7%)	.173
Diabetes mellitus	34 (38.2%)	21 (20%)	.005
COPD	14 (15.7%)	13 (12.4%)	.502
Laboratory results			
Haemoglobin (mmol/L)	7.6 ± 1.1	8.3 ± 1.0	<.0001
eGFR (mL/min/1.73 m^2^)	67 ± 27	73 ± 25	.100

Patient characteristics at the 6-month follow-up. Data presented as mean ± SD or *n* (%). In case of missing data, the number of patients with available data is noted in brackets.

a Subjects can have multiple HF aetiologies. Those categorized as ischaemic are any subject who selected ischaemic, including those with multiple selections.

b Data are a combination of medical history and the first 6 months of follow-up (before the 6MWD).

c Data are based on the first 6 months of follow-up (before the 6MWD).

Abbreviations: 6MWD, = six-minute walk distance; COPD, chronic obstructive pulmonary disease; eGFR, estimated glomerular filtration rate; EQ-5D VAS, EuroQoL-5 Dimensions visual analogue scale (a measure of quality of life); INTERMACS, Interagency Registry for Mechanically Assisted Circulatory Support; LVEF, left ventricular ejection fraction; NYHA, New York Heart Association.

### 6MWD as a predictor of adverse outcomes

Overall median follow-up time was 4.8 [2.3, 5.0] years, patients who walked less than the median distance were followed for 4.9 [2.3, 5.0] years, and patients who walked more than the median for 4.1 [2.2, 5.0] years. During follow-up, 20 of 194 patients with available 6MWD data (10.3%) underwent HTx, compared with 16 of 224 patients without 6MWD data (7.1%, *P* = .30). Among patients with available 6MWD data, transplantation rates were 7.9% (7/89) in the < median group and 12.4% (13/105) in the ≥ median group (*P* = .35). Kaplan-Meier analyses stratified by median 6MWD did not demonstrate significant differences in overall survival (**Figure 1A**, log-rank *P* = .379), survival free from RHF (**Figure 1C**, *P* = .334), or survival free from rehospitalization (**Figure 1D**, *P* = .243), whereas survival free from HRAEs differed significantly, albeit slightly (**Figure 1B**, *P* = .049). Five-year overall survival was 68.0% (95% CI, 56.3-77.2%) in patients with 6MWD < median compared to 73.1% (95% CI, 61.6-81.7%) in those with 6MWD ≥ median. Cox regression models adjusted for age and sex confirmed no significant association between 6MWD (per 10 m increase) and adverse outcomes, including overall mortality (HR 0.98, 95% CI, 0.96-1.01, *P* = .26), survival free from HRAE (HR 0.99, 95% CI, 0.97-1.01, *P* = .34), or survival free from RHF (HR 0.98, 95% CI, 0.95-1.01, *P* = .15). There was no significant difference in overall survival when using the conventional limit of 300 m either (61.9%, 95% CI, 44.8-75.1% in subjects with 6MWD <300 m vs 73.5%, 95% CI, 64.4-80.7% in subjects with 6MWD ≥300 m, *P* = .12, **Figure S6**).

**Figure 1.**
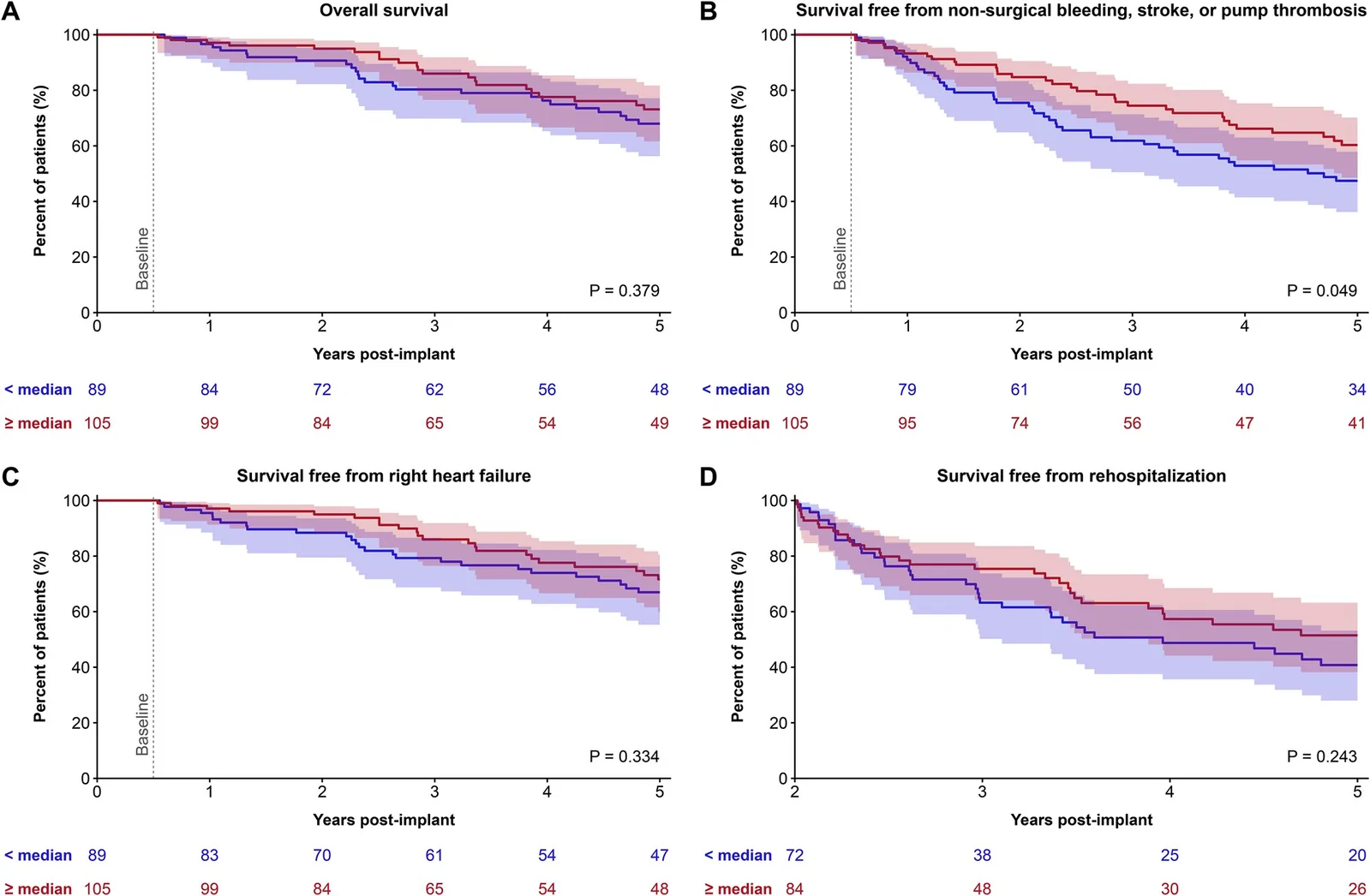
Kaplan-Meier Plots of Survival Outcomes, Stratified by 6MWD at 6 Months Post-Implant. Four Kaplan-Meier plots of (5-year estimates of the 6MWD < median vs ≥ median groups): (A) overall survival (log rank *P* = .379) [68.0% vs 73.1%], (B) survival free from haemocompatibility-related adverse events (log-rank *P* = .049) [47.4% vs 60.3%], (C) survival free from right heart failure (log rank *P* = .334) [67.0% vs 71.6%], and (D) survival free from hospitalization (log rank *P* = .243) [40.8% vs 51.5%]. Please note that rehospitalizations were only recorded 2-5 years from implant.

Apart from infection and bleeding episodes, which are well-known complications of LVAD therapy, adverse event rates were generally low across the cohort (**Table 2**). Patients who walked less than the median 6MWD experienced more adverse events during follow-up, including higher incidences of major bleeding (IRR 2.88, *P* < .001), major infection (IRR 2.14, *P* < .0001, with specifically percutaneous site infections at IRR 1.81, *P* < .001), and renal dysfunction (IRR 4.78, *P* = .033). In contrast, 6MWD was not predictive of arrhythmias (*P* = .242) or haemorrhagic stroke (*P* = .161). With respect to RHF, most events occurred before the 6-month baseline (18 patients), compared with only 5 patients thereafter. A history of RHF was more common in patients walking less than the median distance (15% vs 5%, *P* = .019), and although non-significant, the incidence remained higher from 6 months on (IRR 5.31, *P* = .105).

**Table 2.** Adverse Events, Stratified by Distance Walked </≥ Median (350 m) at 6 Months Post-implant

	Distance < median (*n* = 89) *Follow-up: 284.4 patient years*			Distance ≥ median (*n* = 105) *Follow-up: 302.2 patient years*				
Event	*n* (%)	Events	EPPY	*n* (%)	Events	EPPY	IRR [95% CI]	*P*
Cardiac arrhythmias	8 (9%)	18	0.06	11 (11%)	14	0.05	1.37 [0.68,2.75]	.242
Major bleeding	20 (23%)	46	0.16	13 (12%)	17	0.06	2.88 [1.65,5.02]	**<.001**
Gastrointestinal	6 (7%)	11	0.04	4 (4%)	6	0.02	1.95 [0.72,5.27]	.128
Requiring surgery	4 (5%)	5	0.02	3 (3%)	4	0.01	1.33 [0.36,4.95]	.563
Major infection	53 (60%)	145	0.51	49 (47%)	72	0.24	2.14 [1.61,2.84]	**<.0001**
Inflow or outflow tract	3 (3%)	5	0.02	6 (6%)	7	0.02	0.76 [0.24,2.39]	.770
Localized non-device	28 (32%)	51	0.18	12 (11%)	16	0.05	3.39 [1.93,5.94]	**<.0001**
Percutaneous site	32 (36%)	70	0.25	30 (29%)	41	0.14	1.81 [1.23,2.67]	**<.0001**
Sepsis	13 (15%)	19	0.07	8 (8%)	8	0.03	2.52 [1.10,5.77]	**.015**
Ischaemic stroke	1 (1%)	1	0.00	0	0	0.00	N/A	N/A
Haemorrhagic stroke	8 (9%)	8	0.03	4 (4%)	4	0.01	2.13 [0.64,7.06]	.161
Other neurological dysfunction	6 (7%)	6	0.02	5 (5%)	5	0.02	1.28 [0.39,4.18]	.556
Renal dysfunction	7 (8%)	9	0.03	2 (2%)	2	0.01	4.78 [1.03,22.13]	**.033**
Respiratory failure	5 (6%)	6	0.02	2 (2%)	2	0.01	3.19 [0.64,15.80]	.122
Right heart failure	4 (5%)	5	0.02	1 (1%)	1	0.00	5.31 [0.62,45.48]	.105

N/A = not applicable, as only 1 event was registered in 1 group. Bolded *P*-values indicate statistical significance (*P* < 0.05).

## DISCUSSION

This analysis of the prognostic value of 6MWD measured at 6 months post-HM3 implant in the multicentre ELEVATE registry revealed several important findings.

First, patients walking less than the median distance (350 m) were older, had more comorbidities, and reported lower quality of life, with 6MWD correlating positively with EQ-5D VAS and haemoglobin, and inversely with NYHA class. This was consistent with a previous ELEVATE report.^19^

Second, 6MWD was not independently prognostic of overall survival, survival free from RHF, or rehospitalization, whether analysed as a dichotomous or continuous variable. Survival free from HRAE differed significantly (marginally) between groups when 6MWD was dichotomized at the median (log-rank *P* = .049), although this was not confirmed when 6MWD was modelled as a continuous variable (*P* = .34), and the Kaplan-Meier CIs overlapped throughout follow-up. The relationship between HRAEs and functional capacity may be bidirectional, as early HRAEs may impair postoperative recovery, while preoperative frailty or reduced physiological reserve may predispose patients to both HRAEs and poorer subsequent functional capacity; standardized preoperative frailty assessments were not available to explore these mechanisms. Five-year survival estimates were numerically higher in patients with longer walk distances across all 4 outcomes, but differences were modest and only nominally significant for HRAE-free survival. Previously, a single study has examined the prognostic value of 6MWD in previous-generation axial-flow LVAD recipients.^13^ In their 65-patient cohort with 6MWD at a median 3.6 months from implant, longer 6MWD was associated with improved survival (HR 0.898 per 10 m walked, *P* < .001), while patients walking <300 m (*n* = 20) had worse long-term survival (log-rank *P* = .01). In this analysis, we did not find significant differences in survival between 6MWD < or ≥ 300 m (**Figure S6**) or 6MWD on a continuous scale. Of note, the assist device was different; our cohort was 11 years younger (median), and 6MWD was assessed about 2 months further from implant to account for post-implant rehabilitation.

Third, patients in the < median group experienced higher rates of several adverse events, including major bleeding (IRR 2.88), major infection (IRR 2.14, with percutaneous site infections IRR 1.81), and renal dysfunction (IRR 4.78). These observed associations may reflect the interplay between frailty, comorbidity burden, and functional capacity, given the greater comorbidity burden of this group, along with a trend towards lower renal function at baseline (eGFR 67 ± 27 vs 73 ± 25 mL/min/1.73m², *P* = .10). Although the incidence of RHF was higher in the 6MWD < median group, most RHF episodes occurred before the 6-month landmark, suggesting confounding by early events. The direction of causality for these associations is difficult to establish: patients who ultimately walked shorter distances already had a numerically higher rate of prior driveline infections at baseline, and most RHF episodes occurred before the 6-month landmark (**Table 1**), suggesting that both infection susceptibility and RHF may reflect pre-existing vulnerability rather than being a function of reduced functional capacity.

Finally, 6MWD correlated with both quality of life and NYHA class, supporting a link between functional capacity, patient-perceived health status, and clinician-assessed symptom burden.

Taken together, these findings suggest that while 6MWD post-LVAD-implant reflects patient vulnerability and functional status, it has limited prognostic value for long-term survival in contemporary LVAD recipients.

### Submaximal vs maximal functional assessment

After LVAD implantation, 6MWD improves substantially and remains improved throughout long-term support,^19^^,^^21^^,^^22^ whereas pVO_2_ shows only modest improvement,^14^^,^^23^ remaining severely suppressed in most patients.^11^ This divergence is physiologically plausible: fixed-speed LVADs provide adequate circulatory support during submaximal exertion but cannot proportionally augment flow at maximal effort—instead, patients increasingly rely on haemodynamic reserve at higher loads. Furthermore, residual RV reserve, chronotropic incompetence, anaemia, and/or iron deficiency mostly constrain maximal performance,^14^^,^^15^^,^^24^ while 6MWD is determined predominantly by non-cardiac factors.^15^^,^^19^ A previous study measuring oxygen consumption during 6MWD found that LVAD patients approached maximal exertion (6MWD pVO_2_ was 96% ± 8% of subsequent CPET-derived pVO_2_).^25^ Another study found that, while pVO_2_ and 6MWD remain correlated after LVAD implantation, the slope is steeper (LVAD patients walked ∼80 m farther than non-LVAD HF patients at a given pVO_2_).^16^ Consequently, variability in anaerobic tolerance and comorbidities appears to decouple 6MWD from pVO_2_ and weaken its association with prognosis. As such, pVO_2_ better captures native physiological reserve, including right ventricular, chronotropic, and peripheral function, explaining its stronger association with mortality outcomes.^11^^,^^12^

For strategies aimed at “net-life prolongation”—optimizing LVAD-to-heart transplant (HTx) sequencing by safely deferring some patients while prioritizing others—prognostic tools must accurately discriminate mortality risk. Given our findings, the role of 6MWD appears limited in this regard. While it is a simple measure of functional capacity and correlates with patient-reported outcomes, it did not predict mortality, RHF, strokes, or other major adverse events, which are potentially detrimental to HTx-listing, in our cohort. Thus, the utility of 6MWD in LVAD patients may be confined to descriptive monitoring of functional recovery, whereas prognostic evaluation may be better served by CPET-derived measures. This is completely in line with the current ISHLT guidelines, which recommend 6MWD for functional assessment,^10^ whereas listing criteria for HTx are reserved for pVO_2_ (and other CPET-derived measures).^26^

### Limitations

The main limitation of the ELEVATE registry is the real-world data collection based on each centre’s standard of care, which allowed for missing data when specific assessments were not routinely performed. However, this approach also represents a key strength, as it reflects actual hospital practices in contemporary LVAD management. Six-minute walk distance data were available in 46% of eligible patients. As patients without 6MWD data were older and more frequently classified as INTERMACS profiles 1 or 2 and NYHA class IV (**Table S1**), selection bias may have led to underrepresentation of the most vulnerable patients, potentially attenuating the observed prognostic signal. Overall, the patient characteristics in the ELEVATE registry are typical of those receiving an LVAD in Europe and the Middle East, including demographics (age and sex), preoperative INTERMACS profile, presence of temporary mechanical circulatory support, risk factors for HRAEs, and therapy goal (bridge to transplant and destination therapy). However, HTx rates should be interpreted with caution, given the differing urgency criteria and organ allocation systems across the participating countries, which may influence transplantation rates independently of patient characteristics. Participating centres represented varying implant volumes and different levels of prior experience with the HM3 technology, supporting broad real-world generalizability. While there was no direct source data verification, the entries were monitored remotely and queried to resolve inconsistencies, ensuring a high level of reliability. Despite the extended duration of follow-up, patient withdrawal was uncommon at 5 years.

## CONCLUSION

Shorter 6MWD at 6 months post-implant was associated with higher rates of bleeding, infections, and renal dysfunction, but not survival, freedom from RHF, or rehospitalization. Six-minute walk distance correlated with patient-centred outcomes (quality of life and NYHA class). These findings indicate that 6MWD primarily reflects patient vulnerability rather than long-term prognosis in contemporary LVAD recipients.

## Supplementary Material

ivag241_Supplementary_Data

## Data Availability

The data supporting the findings of this study are available from the corresponding author upon reasonable request.
