# Supplementary material for: Six-Minute Walk Distance and Long-Term Outcomes in Contemporary Left Ventricular Assist Device Recipients: The ELEVATE Registry
Source: Interdiscip Cardiovasc Thorac Surg. 2026 Sep 11;41(11):ivag241. doi: 10.1093/icvts/ivag241 (PMC13730604; doi:10.1093/icvts/ivag241)
Supplement: Supplementary file 1 [file ivag241_supplementary_data.docx]

# Supplementary material

### Supplementary Table S1 – Patient characteristics, stratified by 6MWD availability at the six-month follow-up

|  | **6MWD performed (n = 194)** | **6MWD not performed (n = 224)** | **p** |
| --- | --- | --- | --- |
| Age (years) | 54.0 ± 12.3 | 56.7 ± 11.2 | 0.021 |
| Female sex | 27 (13.9%) | 20 (8.9%) | 0.107 |
| Caucasian | 145 (75.1%) | 173 (78.3%) | 0.449 |
| Body mass index (kg/m^2^) | 27.5 ± 4.9 | 27.0 ± 5.1 | 0.304 |
| Mean arterial pressure (mmHg) | 79 ± 11 | 76 ± 11 | 0.017 |
| LVEF (%) | 19 ± 6 | 18 ± 6 | 0.184 |
| Hemoglobin (mmol/L) | 7.8 ± 1.5 | 7.5 ± 1.4 | 0.020 |
| eGFR (mL/min/1.73m^2^) | 65 ± 26 | 59 ± 31 | 0.049 |
| Intent |  |  | 0.263 |
| Bridge to transplant | 121 (62.4%) | 153 (68.3%) |  |
| Bridge to recovery | 6 (3.1%) | 4 (1.8%) |  |
| Destination therapy | 53 (27.3%) | 59 (26.3%) |  |
| Other | 14 (7.2%) | 8 (3.6%) |  |
| INTERMACS profile |  |  | 0.146 |
| 1 | 16 (8.2%) | 23 (10.3%) |  |
| 2 | 34 (17.5%) | 59 (26.3%) |  |
| 3 | 75 (38.7%) | 82 (36.6%) |  |
| 4–6 | 66 (34.0%) | 57 (25.5%) |  |
| Not provided | 3 (1.5%) | 3 (1.3%) |  |
| NYHA class |  |  | 0.017 |
| I or II | 0 | 0 |  |
| IIIa | 26 (13.4%) | 14 (6.3%) |  |
| IIIb | 75 (38.7%) | 70 (31.3%) |  |
| IV | 89 (45.9%) | 121 (54.0%) |  |
| Not provided | 4 (2.1%) | 19 (8.5%) |  |
| 6MWD pre-implant | 140.5 ± 147.3 | 50.6 ± 113.5 | <0.0001 |
| EQ-5D VAS score | 37.2 ± 20.6 | 32.2 ± 18.0 | 0.066 |
| History of Diabetes | 28.4% (55/194) | 22.8% (51/224) | 0.191 |
| History of COPD | 13.9% (27/194) | 6.7% (15/224) | 0.014 |
| History of AFib | 43.3% (84/194) | 37.9% (85/224) | 0.776 |
| Right heart catheterization |  |  |  |
| CVP (mmHg) | 10 ± 6 | 12 ± 7 | 0.019 |
| Systolic PAP (mmHg) | 50 ± 14 | 53 ± 17 | 0.083 |
| Diastolic PAP (mmHg) | 24 ± 8 | 27 ± 10 | 0.005 |
| Mean PAP (mmHg) | 34 ± 10 | 36 ± 12 | 0.037 |
| Cardiac output (L/min) | 3.8 ± 1.0 | 3.8 ± 1.2 | 0.876 |
| Cardiac index (L/min/m^2^) | 1.87 ± 0.48 | 1.93 ± 0.62 | 0.369 |
| PVR (dynes/sec/cm^-5^) | 307.7 ± 753.0 | 514.1 ± 1795.2 | 0.205 |

Only patients alive at the six-month follow-up were included. Please note that patients who did not perform a 6-minute walk distance at six months due to severe HF symptoms were imputed as zero meters and included in the “6MWD performed” group. Abbreviations: 6MWD = six-minute walk distance, CVP = central venous pressure, eGFR = estimated glomerular filtration rate, EQ-5D VAS = EuroQoL-5 Dimensions visual analogue scale (a measure of quality of life), INTERMACS = Interagency Registry for Mechanically Assisted Circulatory Support, LVEF = left ventricular ejection fraction, NYHA = New York Heart Association, PAP = pulmonary artery pressure, PVR = pulmonary vascular resistance

### Supplementary Figure S1 – Distribution of six minute walk distances at the six month follow-up

### Supplementary Figure S2 – Kaplan-Meier plot of overall survival stratified by availability of 6MWD at six months

Five-year survival in patients with 6MWD (+ 6MWD): 70.7% (95% CI: 62.8-77.3%) vs. 67.4% (95% CI: 60.1-73.7%) in those without (− 6MWD).

### Supplementary Figure S3 – Relationship between 6-minute walk distance (6MWD) and EQ-5D visual analogue scale score at six months

Pearson’s r = 0.2277

### Supplementary Figure S4 – Relationship between 6-minute walk distance (6MWD) and hemoglobin at six months

Pearson’s r = 0.19854

### Supplementary Figure S5 – Relationship between 6-minute walk distance (6MWD) and NYHA class at six months

Pearson’s r = -0.47112

### Supplementary Figure S6 – Kaplan-Meier plot of overall survival stratified by 6MWD relative to 300 meters

Five-year survival in patiendatts with 6MWD <300m 61.9% (95% CI: 44.8-75.1%) vs. 73.5% (95% CI 64.4-80.7%) in those with 6MWD ≥300m.
